# Treatment of Iron-Loaded Veterinary Sarcoma by *Artemisia annua*

**DOI:** 10.1007/s13659-014-0013-7

**Published:** 2014-04-12

**Authors:** Elmar Breuer, Thomas Efferth

**Affiliations:** 1 Veterinary Clinic for Pets, Müllheim/Baden, Germany; 2 Department of Pharmaceutical Biology, Institute of Pharmacy and Biochemistry, Johannes Gutenberg University, Mainz, Germany

**Keywords:** Artemisinin, Asteraceae, Scopoletin, Veterinary cancer, Comparative oncology

## Abstract

Artemisinin, a constituent of *Artemisia annua* L., is a well-known antimalarial drug. Artemisinin-type drugs also inhibit cancer growth in vitro and in vivo. Herbal extracts of *A.annua* inhibit the growth of cancer cell lines. Here, we report on the use of capsules containing powder of *Herba Artemisiae annuae* to treat pet sarcoma. The surgical tumor removal as standard treatment was supplemented by adjuvant therapy with *A.annua*. One cat and one dog with fibrosarcoma survived 40 and 37 months, respectively, without tumor relapse. Two other dogs suffering from fibrosarcoma and hemangioendothelial sarcoma also showed complete remission and are still alive after 39 and 26 months, respectively. *A.annua* was well tolerated without noticeable side effects. These four cases indicate that *A.annua* may be a promising herbal drug for cancer therapy.

## Introduction

*Artemisia annua* L. is a medicinal plant used in traditional Chinese medicine to treat fever and chills. In the late 1960 and 1970 s, Chinese scientists discovered that artemisinin, one of the constituents of *A. annua,* is active against malaria [1, 2]. The anti-malarial activity of artemisinin was confirmed by many scientists worldwide. Nowadays, derivatives of artemisinin such as artemether and artesunate are well-established drugs in the fight against malaria. In the 1990 s, the pioneering work by us and others showed that artemisinin and its derivatives inhibit cancer growth in vitro [3–7]. In subsequent years, it turned out that artemisinin-type compounds are able to kill cell lines of many different tumor types, *e.g*. leukemia, lymphoma, melanoma, brain tumors, carcinoma of the colon, breast, ovary, lung, kidney, and many others [8–10]. Investigations into the mode of action revealed that artemisinin may form active radical oxygen species and carbon-centered radical molecules leading to oxidative stress [11–15], DNA damage [16, 17], adduct formation of specific target proteins [18, 19], cell cycle arrest [20], interaction with signal transduction pathways [21–26], induction of apoptosis and autophagy [7, 27–31], and inhibition of angiogenesis [32–35]. Artemisinin-type drugs have also been shown to act against cancer in vivo using transplantable murine syngeneic tumors [6, 36–38] and human xenograft tumors [32, 39–47]. The clinical efficacy has been described in several reports on the use of these compounds [48, 49] and more recently in clinical trials in veterinary tumors (spontaneous tumors in dogs) and human cervical carcinoma [50, 51].

Whereas artemisinin and its derivatives are well analyzed both in malaria and cancer, less is known about the bioactivity of plant extracts of *A.annua*. The antimalarial activity of *A.annua* tea or extracts has been investigated in animal experiments [52, 53]. Malaria treatment with *A.annua* tea is being widely used in Africa by the indigenous population outside official health care systems [54–60]. Whether or not *Herba Artemisiae annuae* is active against cancer in vivo is unknown, although cytotoxic activity in vitro has been reported [61, 62].

Here, we report on the use of *Herba A.annuae* in pets suffering from sarcoma. Dogs and cats may spontaneously develop cancer, making them to suitable subjects for pharmacological intervention [63]. Treatment of tumors in three dogs and one cat showed a considerable response of tumors to the herbal preparation.

## Materials and Methods

### Material

*Artemisia annuaL*. preparations (Luparte^®^) were obtained from Lupovet GmbH (Müllheim/Baden, Germany). Using a semiautomated device 450 or 150 mg of the 1:30 extracted and pulverized *Herba A. annuae* were for technical reasons enriched with 50 or 17 mg Inulin and enclosed in capsules size “O” or “4”, respectively.

### Thin Layer Chromatography (TLC)

The method was performed as described with modifications [64]. The test solution was prepared by dissolving 3 g *Herba A. annuae* powder in 50 mL petroleum ether preheated to 60–90 °C, refluxing for 1 h and filtering. The filtrate was evaporated to dryness and the resulting residue was dissolved in 30 mL hexane. The solution was partitioned with 20 % acetonitrile–water twice or trice and the combined acetonitrile phases were evaporated to dryness. The evaporated dry residue was dissolved in 0.5 mL ethanol. Artemisinin and scopoletin (each 1 mg/mL) were used as reference substances (Sigma-Aldrich, Taufkirchen, Germany).

The thin layer chromatography was performed using Silica gel 60 F254 plates (Merck, Darmstadt, Germany), onto which 5 µL each of test solution and reference substances were spotted to the Silica gel plates. The plates were developed in a chromatographic jar containing mobile phase composed of cyclohexane/ethylacetate/glacial acetic acid in ratios of 20:10:1. After developing the plate was air dried examined on day light, UV-light (365 nm) and sprayed with spray reagent composed of 5 mL anisaldehyde, 10 mL glacial acetic acid mixed with 85 mL methanol and concentrated H_2_SO_4_

### Patients

One female and two male dogs as well as one male cat between 10 and 13 years were diagnosed with hemangioendothelial sarcoma or fibrosarcoma at the Veterinary Clinic, Müllheim. All four animals suffered from a grade 3 or 4 tumor at diagnosis (Table 1).

**Table 1:** Clinical data of animals

	Case 1	Case 2	Case 3	Case 4
Species	cat	dog	dog	dog
Breed	European	Bernese mountain dog	Gordon Setter	Bernese mountain dog
Gender	male	male	female	male
Date of diagnosis	Sept 14, 2011	Dec 28, 2011	Oct 18, 2010	Nov, 25, 2011
Age at diagnosis	10 years	10 years	10 years	10 years
Initial Serum Iron (µg/dL)	222	107	85	124
Histology	fibrosarcoma	fibrosarcoma	hemangioendothelial sarcoma	fibrosarcoma
Staging, Grading:	T3N0M0	T3NxM0	T4N0M0	T3NxM0
Response	complete remission	complete remission	complete remission	complete remission
Survival time (months)	40^a^	37^a^	39^a^	26^a^
Status	dead	dead	alive	alive

^a^ at Febr 10, 2014

After the decision was made to use Luparte^®^, the serum iron (normal range between 140 and 170 µg/dL) was measured. Between blood taking and getting back the serum iron results from the clinical diagnosis laboratory, iron was given *p.o*. b.i.d or intramuscularly every 3 days to mark the iron affine malignant cells. The initial “blind” dose of orally given iron (*e.g*. Ferrosanol^®^ capsules 100 mg) was about 100 mg/30 kg b.i.d. or about 100 mg/10 kg weight Ursoferran i.m. two times a week. The iron application was continued for the entire treatment time, and was attuned to maintain the iron level at 250 ± 30 µg/dL.

From the fourth day onward, the animals were treated *p.o*. two to three times daily with one capsule (150 mg in the cat, 450 mg in the dogs) of *Herba A. annuae* simultaneously.

## Results

### Thin Layer Chromatography

**Fig. 1:**
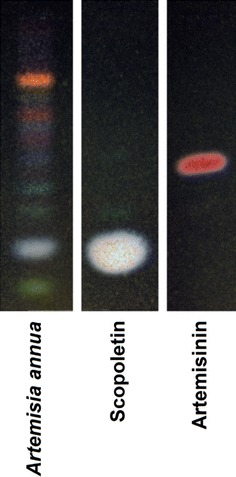
Thin layer chromatography of Herba Artemisiae Annuae (Luparte^®^). Scopoletin and Artemisinin were used as reference compounds

As a starting point, we analyzed the composition of the *A. annua* powder used for the preparation of the Luparte^®^ capsules by TLC. As shown in Fig. 1, artemisinin was a minor constituent with an *R*_f_ value resembling the authentic sample *R*_f_ and scopoletin was the abundant compound in the *A. annua* powder. Capsules filled with this plant powder were used for the therapy of animals suffering from sarcoma in an adjuvant setting together with initial surgery.

### Case 1

**Fig. 2:**
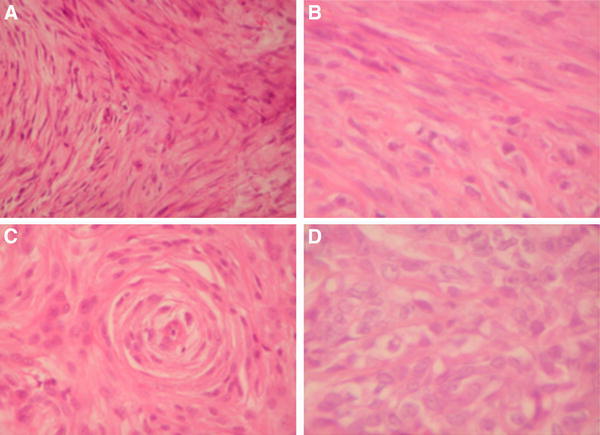
Histology of pet sarcoma. **a** Highly malign fibrosarcoma of the cat case 1 (× 400) **b** Fibrosarcoma of the Bernese mountain dog Case 2 (× 600) **c** Hemangiosarcoma of the Bernese mountain dog Case 3 (× 400) **d** Fibrosarcoma of the Bernese mountain dog Case 4 (× 600)

A 10-year old cat was presented on September 14th, 2011 with a highly malignant fibrosarcoma subcutaneously localized at the lower front flank, which infiltrated muscles and fat tissue and invaded veins (Fig. 2a; Table 1). The tumor did not tend to grow in a capsule-like morphology and revealed marginal fiber formation. Concomitant inflammation with lymphoplasmacellular dominance and lymphofollicular proliferation were detectable. The tumor was removed by surgery and adjuvantly treated with *A. annua* (3 capsules 150 mg/day) without iron application due to high serum iron values. No side effects were observed. Euthanasia was performed 40 months later on January 7th, 2014 because of chronic hepatosis. The tumor was not detectable clinically or by sonography at that time.

### Case 2

A buccally localized, highly malignant mesenchymal neoplasia mainly of fibrosarcomatous architecture was diagnosed in a 10-year old dog (Bernese mountain dog mix) on December 28th, 2010 (Fig. 2b; Table 1). The highly proliferating tumor showed tuberous infiltration. Areas of fibroblast proliferation were interspersed with capillary shoots. A massive plasmacellular neutrophilic inflammation with a high portion of mast cells was superimposed on the tumor. The tumor was incompletely removed via surgery and treated with *A. annua* (2 capsules 450 mg/day) in an adjuvant manner from the fourth day on after marking the malignant cells with iron from the Ferrosanol 100 mg capsules. No side effects were observed. The dog was euthanized 37 months later on March 20th, 2012 because of uremia. At this time no tumor relapse was detected.

### Case 3

A 10-year old Gordon Setter with a clinically obvious breast cancer with a length of about 40 cm and a width of about 10 cm was presented with highly acute course and massive concomitant edematous inflammation October 18th, 2010. The tumor was diagnosed as hemangioendothelial sarcoma with multiple foci (Fig. 2c; Table 1). Capsule formation took place partially. The tumor foci revealed infiltrating fibrotic nodes. Two sections of lymph nodes with hemosiderosis did not feature metastases. The tumor was operated on and treated with *A. annua* (Luparte^®^) (3 capsules à 450 mg/day) in an adjuvant manner from the fourth day after iron identification marked the remaining malignant cells. The Artemisia capsules were well tolerated without side effects. Thirty-nine months later, by a phone call in the middle of January 2014, we learned that the dog is doing well.

### Case 4

A rapidly growing, irregularly confined tarsal tumor was diagnosed in a 10-year old Bernese mountain dog on November 25th, 2011. Pathological examination revealed that the tumor was a highly malignant fibrosarcoma infiltrating in and fat tissue and invading veins (Fig. 2d; Table 1). The tumor did not form a capsule and revealed marginal fiber formation. Concomitant inflammation with lymphoplasma cellular dominance and lymphofollicular proliferation was detectable. The fibrosarcoma was surgically removed, a skin mesh was transplanted on the remaining defect and the dog was treated two times daily with capsules containing 450 mg each for about 2 years. No side effects appeared. Twenty-six months later, the dog is still healthy without any signs of relapse.

## Discussion

In the present investigation, we addressed the question of whether or not an *A. annua* preparation is able to inhibit tumors in vivo. This project was inspired by data we and others collected, which indicated that *A. annua* extracts in addition to isolated artemisinin, may inhibit cancer cells in vitro[5, 61, 65]. Nevertheless, the question of whether or not the cytotoxic activity observed in vitro can be translated to living organisms remains. In many cases this is unusual. The observations we made with a 1:30 *A.annua* extract for the treatment of spontaneous tumors and dogs and cats are encouraging for starting larger placebo-controlled and randomized clinical trials. Fibrosarcoma and hemangioendothelial sarcoma are tumor types, which are primarily treated by surgical removal. Survival times for dogs with fibrosarcoma treated with surgery range from 7 to 12.2 months. Adding adjuvant radiotherapy improved outcomes by an average of 743 days [68]. Radiotherapy of sarcoma in dogs resulted in survival times between 8 and 19.7 months [69–74], whereas the success of chemotherapy is limited [75]. This is a non-satisfying situation and novel treatment strategies are urgently required. Here, we report on four pets suffering from fibrosarcoma or hemangiosarcoma treated by surgery plus *A.annua* capsules after the remaining malignant cells were marked with iron. The combined use of surgery and adjuvant treatment with iron and *A.annua* resulted in survival times between 26 and 40 months, and two of the four animals are still alive. This indicates that adjuvant iron-triggered *A.annua* impacts the overall survival rates of dogs suffering from sarcoma and improves the treatment success over that of surgery alone. Controlled clinical trials are warranted to verify these first experiences with iron-triggered *A.annua* in pet cancer therapy. The comparative relevance of this phytochemical trial to human oncology remains to be seen. As of now, no comparable alternative is known to the authors.

While there are great efforts to test drugs in vivo in mice, the value of veterinary tumors as a comparative in vivo test is frequently underestimated. Most commonly, murine syngeneic or human xenograft tumors transplanted into mice are used to test the activity of cytotoxic compounds in vivo. Both model systems have advantages and disadvantages [66]. Syngeneic and xenograft tumors are easy to maintain and well-established tumor lines are available. They are usually maintained intraperitoneally or subcutaneously, but these are sites that may not be relevant for the clinical situation. In addition, human xenografts are transplanted into athymic nude mice, which lack an intact immune system. More relevant tumor models such as orthotopic tumors require surgical manipulation and are, thus, time-and cost-consuming. In addition, the animals require more care. Therefore, experimentally induced or spontaneously arising veterinary tumors may represent a promising option [63, 66, 67]. Our own experience with *A. annua* and recently published data with artesunate in dog tumors [51] favorable suggest that veterinary tumors are a suitable model to test the anticancer activity of drugs in vivo, before applying them to human cancer patients. In this way, such trials provide “comparative oncology”.

An interesting result was that artemisinin represented only a minor constituent in *A.annua* and that scopoletin was the most abundant phytochemical. It is well known that *A.annua* contains a large panel of different secondary metabolites, including scopoletin, which are cytotoxic [5, 57, 76–79]. The activity of the *A.annua* preparation in pet tumors and the presence of large amounts of scopoletin raise the question of whether scopoletin might contribute to the anticancer effect of this plant rather than artemisinin. The in vitro cytotoxicity of scopoletin towards cancer cells has been previously described [80–82].

In conclusion, the results of the present investigation give hope that approaches with *A.annua* may be promising for the treatment of veterinary tumors. Furthermore, the analysis of scopoletin and other hydroxylated and methoxylated flavonoids as a therapeutic principle in *A.annua* deserves further attention.
